# Applications of reflectance confocal microscopy in photoaging and aesthetic conditions: skin characterization and treatment monitoring

**DOI:** 10.1007/s00403-025-04020-5

**Published:** 2025-03-08

**Authors:** Gaurav N. Pathak, Aarushi K. Parikh, Aleena Saifullah, Hamza Ajmal, Madeline Tchack, Noah Musolff, Babar K. Rao

**Affiliations:** 1 Department of Dermatology, Rutgers Robert Wood Johnson Medical School, Somerset, NJ USA; 2 Department of Dermatology, University of Louisville, Louisville, KY USA; 3 Department of Dermatology, Rao Dermatology, Atlantic Highlands, NJ USA; 4 Rocky Vista University College of Osteopathic Medicine, Parker, CO USA

**Keywords:** Reflectance confocal microscopy, RCM, Aesthetic, Color cosmetics, Polymers, Formulation/stability

## Abstract

Reflectance confocal microscopy (RCM) is an in-vivo, non-invasive imaging modality that provides a high-resolution image of the epidermis and upper dermis. RCM has been utilized as a diagnostic aid for several inflammatory, infectious, and malignant skin conditions; however, its use for clinical and aesthetic skin purposes has not been well established. The purpose of this review is to describe the landscape of RCM utilization for the application of aesthetic skin conditions. A comprehensive literature search was conducted using PubMed using the search terms “reflectance confocal microscopy cosmetic”, and “reflectance confocal microscopy aesthetic”. The search was limited to clinical and animal studies published in English in the last 10 years. RCM must have been utilized to measure an aesthetic dermatological outcome to be eligible for the review. After data abstraction, a total of 46 studies that met the inclusion criteria were identified. The most common utilization of RCM for cosmetic conditions included treatment monitoring and skin morphologic characterization. The primary skin conditions evaluated included skin aging, pigmentation, skin dryness, irritated, and sensitive skin related conditions. Treatment monitoring was primarily conducted for topical agents for skin hydration, skin UV protection, acne, skin dryness, and skin pigmentation purposes. Identification of histo-structural correlations with aesthetic skin conditions may pave the way for future aesthetic drug development. As the popularity of cosmetic dermatologic procedures continues to increase, utilization of RCM for skin characterization and treatment monitoring may be beneficial.

## Introduction

Reflectance Confocal Microscopy (RCM) is a noninvasive imaging modality for assessing the skin, that provides a horizontal view from the surface of the skin down to the superficial dermis, via focused illumination of light at specific skin morphologic structures [1]. The methodology involves concentrating light through an objective lens onto a spot on the sample through a detection pinhole, with a subsequent collection of the reflectance signal. The resulting images develop from variations in refractive indices of skin components due to light backscattering. Currently, two RCM probes are prominently employed: the static RCM (Vivascope 1500) and a handheld RCM device (Vivascope 3000), both contributing to the evolving landscape of dermatologic diagnostics.

The current clinical applications and FDA-approved indications for RCM predominantly focus on its utilization in the evaluation of skin tumors, inflammatory, and infectious conditions [2–4]. It has been successfully characterized for a variety of dermatologic conditions, including eczema, psoriasis, fungal conditions, scabies and demodex mites. RCM’s serves as a diagnostic aid, enhancing both specificity and sensitivity in diagnosing skin conditions as well as mapping margins during cancer resections and excisions [5]. However, studies assessing RCM in the context of cosmetic and aesthetic conditions are limited. As the number of cosmetic dermatology visits continue to increase, utilization of this promising, non-invasive imaging method may enhance treatment efficacy monitoring and reduce the need for biopsy. The aim of this review is to describe the landscape of RCM utilization in the application of aesthetic skin conditions.

## Methods

A comprehensive medical literature review to evaluate the aesthetic applications of reflectance confocal microscopy (RCM) using PubMed was performed. PubMed search terms utilized included “reflectance confocal microscopy cosmetic”, and “reflectance confocal microscopy aesthetic”. The search was limited to publications of randomized clinical trials, observational studies, case reports, case series, and animal studies that were published within the last 10 years. RCM must have been used in some capacity to measure an aesthetic dermatological outcome in vivo. Certain conditions such as acne, and atopic dermatitis, have significant overlap in medical and cosmetic practices and were both eligible for this review.

Literature reviews, systematic reviews, scoping reviews, studies not in English, studies missing data, and studies without full access were excluded. The parameters of interest were study name, study type, sample size, aesthetic condition, intervention details, RCM measure assessed, RCM related results, and key study conclusions.

## Results

After initial abstraction, a total of 46 studies met the inclusion criteria (Table 1). Majority of the studies evaluated RCM for treatment monitoring in aesthetic skin conditions (Table 2), and the remaining studies primarily characterized morphological changes in cosmetic skin conditions (Table 3).

**Table 1:**
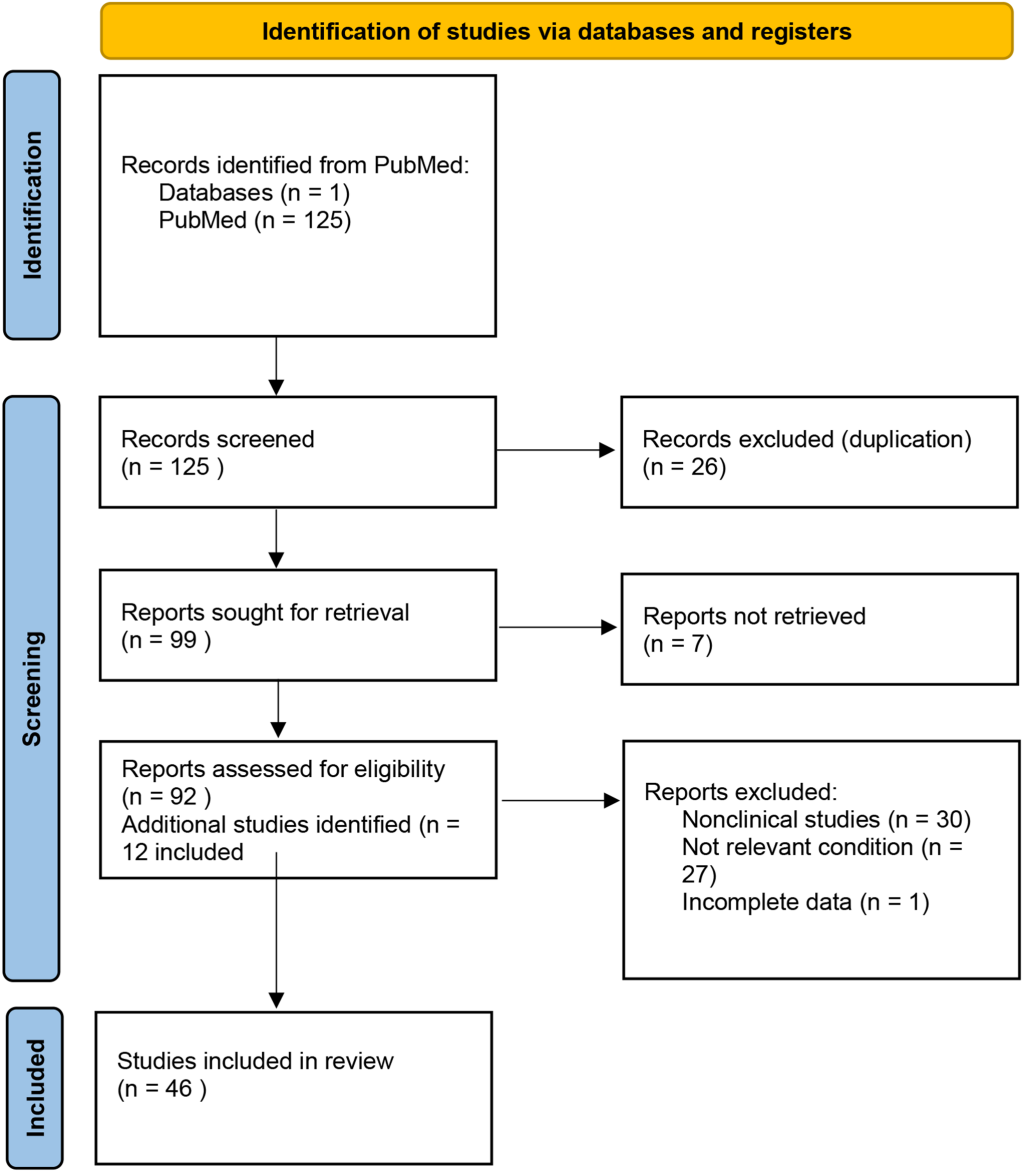
Summary of search strategy for eligible studies

*Consider, if feasible to do so, reporting the number of records identified from each database or register searched (rather than the total number across all databases/registers)

**If automation tools were used, indicate how many records were excluded by a human and how many were excluded by automation tools

Source: Page MJ, et al. BMJ 2021;372:n71. doi: 10.1136/bmj.n71

This work is licensed under CC BY 4.0. To view a copy of this license, visit https://creativecommons.org/licenses/by/4.0/

**Table 2:** Summary of studies evaluating RCM for treatment monitoring in cosmetic applications

Study	Study type	Sample size	Condition	Intervention	RCM measure assessed	RCM related results	Key study finding
Infante et al. [6]	Prospective observational cohort	10	Healthy skin and hair	Two formulations based on starches and PEG-75 lanolin containing or not containing a Spirulina maxima dry extract	Skin hydration, distance between stratum corneum furrows, morphological and structural characteristics of stratum corneum (brightness) and epidermis, hair quality	Improvement of stratum corneum morphology, increased structure of epidermis granular layer, reduction in furrow distance observed 30 min after application of formulation (*p* < 0.001)	RCM revealed benefits in film forming effect of topical starch + PEG 75 lanolin both with and without Spirulina maxima dry extract
De Molo et, al [7]	Prospective observational cohort	12	Skin quality/film forming effect	Gel and emulsion formulations containing both Kappaphycus alvarezii and Caesalpinia spinosa extracts	Morphological and structural characteristics of the epidermis and SC were measured using the following parameters: brightness, residues, furrow morphology, furrow width and regularity of the skin surface	RCM revealed reduction of furrow width between islands of keratinocytes on the surfaces of the skin, as well as formation of film on skin surface, and brighter appearance of the skin surface	RCM evaluation of two active ingredients Kappaphycus alvarezii and Caesalpinia spinosa revealed a significant film formation that resulted in a reduction in trans-epidermal water loss and skin peeling; however, results were more pronounced in the emulsion formulation.
Kakuda, et.al [8]	Prospective observational cohort	50 people	Healthy skin	Sunscreen (mean SPF 45.6) Two formulations: F1 (sunscreen) and F2 (sunscreen added to biopolymer)	Furrow size, Furrow’s morphology, reflectance of SC, inter keratinocyte brightness	Improvement of furrow morphology and size reduction observed 2 and 4 h after F1 application, and 2,4,6 h after F2 application; Increased SC thickness (*p* < 0.05), increase in inter keratinocyte reflectance (*p* < 0.05)	A film-forming property was observed in RCM images after formulation application, which was more pronounced for F2
Sun et al. [33]	Cohort study	10	Skin rejuvenation	Intense pulsed light treatment	Thickness of SC, minimal thickness of epidermis, thickness of basal layer, density of dermal papillae, and mean diameter of papillae capillaries	SC thickness increased from 4.80 ± 1.48 μm micrometers before treatment to 5.50 ± 1.35 micrometers (*p* < 0.322); minimal thickness of epidermis and thickness of basal layer increased (*p* < 0.002 and 0.018), and dermal papillae density was also increased (0.035)	IPL treatment is a highly effective treatment for skin rejuvenation, and results can be evaluated using RCM imaging.
Royo de la Torre et al[19]	Comparative, double blind trial	119	Nasolabial folds	Follow up large-gel hyaluronic acid (HA) filler and HA + lidocaine	Skin rejuvenation and histological findings	Increased skin rejuvenation with 32% increase in DEJ (*p* < 0.001)	RCM shows histological improvement in DEJ following dermal filler use
Manfredi, et al [27]	Randomized controlled trial	10	Skin dryness	Group 1: Petrolatum moisturizer Group 2: commercially available emulsion moisturizer	Furrow size and morphology, inter keratinocyte reflectance, scales, skin surface irregularity, non-rimmed dermal papillae, exocytosis, dermal inflammation and collagen type	Increased capacitance in both groups, scale score decreased (*p* < 0.001), slight decrease furrow size and epidermal irregularity scores decreased (*p* < 0.001), inter-keratinocyte reflectance increased (*p* < 0.001), no changes in collagen, inflammation and alteration of DEJ noted	RCM allows for evaluation and treatment monitoring of morphologic changes of dry skin
Neves et. al [21]	Prospective observational cohort	40	Skin aging and pigmentation	Topical antioxidant containing 15% L-ascorbic acid, neohesperidin, Pinus pinaster bark, tocopherol, and hyaluronic acid (HA)	SC and epidermal thickness, basal membrane thickness	Increased SC thickness (*p* = 0.047) and morphology with higher surface regularity (*p* = 0.004). Increased thickness of epidermis and improved dermis morphology. Reduction of basal membrane pigmentation (*p* < 0.005).	Serum effectively reduced signs of skin aging, and RCM imaging evidenced the improvement of epidermal and SC morphology.
Mondon, et. al [22]	Prospective cohort study	22	Skin aging	Various anti-aging peptides	Analysis of structural changes, and upper dermal fiber structures	Depth of papillary dermis was reduced from 108 to 63 μm (R²=0.517) dermal fiber circumference improved + 13.2% (*p* < 0.01), subepidermal low-echogenic band decreased by 11% inner face (*p* < 0.01) and 14.4% external surface of forearm (*p* < 0.01)	RCM can detect changes in upper dermis following anti-aging peptide applications
Bencini et al. [18]	Prospective observational study	18	Aged neck skin	Six laser treatments in 4-week intervals using 540-nm erbium-glass fiber laser	Structural changes noted by RCM	Three months after last session, improvement in dyschromia (*p* = 0.0002), wrinkles *p* = 0.0004) and no changes in skin laxity.	RCM can show structural changes of aged neck skin following laser skin resurfacing
Hugo Infante, et al. [15]	Randomized controlled clinical trial	40	Photoaging	2% pure essential oil and 2% pure essential oil in nanoemulsion	Skin hydrolipidic and morphological characteristics	An increased in epidermal thickness (*p* = 0.004) and papillary depth (*p* = 0.03) in essential oil group vs. placebo.	RCM can be utilized to characterize collagen fiber quality and keratocyte cells
Alarcon, et al. [58]	Prospective observational study	10	Photoaging	Irradiation with UVB and UVA before and 8 h after topical cream	In vivo assessment of changes to the skin for signs of photodamage	Significant changes in presence of small bright epidermal cells (*p* = 0.007) and parakeratosis (*p* = 0.001). In photoprotected skin, there was not observed (spongiosis, microvesicles, blood vessel dilation) (*P* < 0.05)	RCM was able to visualize morphological changes of acute UVR including sunburn cells, microvesicles, spongiosis, blood vessel dilation and inflammation.
Harmelin et al. [32]	Prospective randomized comparative trial	22	Abdominal striae	Group 1: no treatment Group 2: Bipolar radiofrequency (rf) with infrared light Group 3: fractional bipolar rf Group 4: Treatment with IR light then fractional bipolar rf	Confocal modifications in the skin following treatment	Baseline: Abnormal elongated dermal papilla shape. Parallel orientation of collagen bundles Post treatment: Rounder dermal papilla, more reticulated collagen fiber arrangement. Differences were noted in group groups 2 and 3 (*p* = 0.02)	RCM allows for visualizations of dermal papilla and collagen bundle shapes following fractionated bipolar radiofrequency and infrared light treatment.
Gomes et. al[16]	Open-label, prospective, control pilot study	10	Ultraviolet damage	Daily protective moisturizer with High UVB and UVA Photoprotection	Sunburn cells, microvesicles, spongiosis, blood vessel dilatation, and inflammation	Reduced appearance of UV damage relating to spongiosis, microvesicles, sunburn cells and blood vessel dilation	Skin UV damage following treatment can be monitored and evaluated RCM technology
Goberdhan et. al[20]	Prospective observational cohort	23	Facial photodamage	0.5% retinol to be applied once every other day for first week then increased frequency as tolerated	Epidermis appearance, SC compactness, abundance of dermal fibers, keratinocyte contour (normal or blurred), pigmented keratinocytes at the DEJ, inflammatory cells in the dermis, dilated vessels.	Decrease in SC thickness observed at 4, 8, and 12 weeks (*p* < 0.02); 5.3% overall decrease in epidermal thickness, increase in number of subjects with uncompact SC. Increase in presence of fibrillar collagen at weeks 8 and 12 (all *p* ≤ 0.0469); decrease in presence of huddled collagen at week 12 (*p* < 0.0313). 18% non-significant decrease in coarse collagen fibers	RCM can be used to objectively assess improvements in skin following retinol use for moderate facial photodamage
Cameli, et al. [38]	Prospective cohort study	20	Solar lentigines	Chemical sunscreens SPF 50 applied daily	Keratinocyte and histopathologic changes of the epidermis	Baseline: Pigmented keratinocytes and increased pigmentation at DEJ 12 weeks post treatment: absence of bright keratinocytes and reduction of periadnexal brightness	RCM can show change in keratinocyte pigmentation post treatment
Arginelli et al. [37]	Cohort study	36	Solar lentigo	Dermo-cosmetic lightening product	DEJ de-structuring score, papillary brightness	Reduction in papillary brightness in post treatment compared to the moisturizing product (*p* < 0.03); there were differences in DEJ destruction and papillary contrast (both *p* < 0.03)	Dermo-cosmetic lightening products reduced lesion hyperpigmentation.
Peng et. al[33]	Prospective observational cohort	43 (22 with irregular borders and 21 with smooth borders)	Pigmented lesions: CALMs	3 sessions of QSAL	Rete peg length and papillae abundance	RCM revealed that in comparison to the smooth bordered lesions, CALM with irregular border presented with shorter rete pegs and less papillae (*p* < 0.05) following laser treatment.	RCM revealed an inverse relationship between response treatment and length/abundance of ret pegs along with papillae.
Cantellia et. al[36]	Clinical trial	40	Melasma	Twice daily application gel containing glabridin, andrographolide, and apolactoferrin for 6 months	Melasma Morphologic structure, dendritic cells	Refractile cobblestone pattern in all patients at baseline and absent in 47.5% of patients post treatment with low and medium in 45% and 7.5% (*p* < 0.001). Dendritic cells reduced from 67.5–10% post treatment (*p* < 0.001*)*	RCM imaging supports the gel’s ability to improve melasma through reduction of cobblestone pattern and reduction in dendritic cell presence.
Cantelli et. al[35]	Clinical trial	​​12	Melasma	Twice daily application of skin whitening serum in combination with SPF50 + sunscreen	Inflammatory infiltrate, melanophage, and dendritic cell abundance	Reduced inflammatory infiltrate, melanophage, and dendritic cell abundance post treatment	RCM revealed significant improvement in hyperpigmentation following 12 weeks of combination skin whitening and sunscreen treatment
Fossa Shirata et al. [17]	Clinical trial	32	Hyperpigmentation	Malar region application of: vehicle, vehicle + ascorbyl tetraisopalmitate (ATIP), vehicle plus spirulina, and vehicle with hydroxytyrosol-titrated olive extract	Skin pigmentation by measuring basal layer brightness	A significant reduction in number of hyperreflective pixels in the basal layer after 42 days of use of the formulations containing ATIP, Spirulina and olive extract	RCM can be useful in assessing changes in pigmentation after use of topical antioxidant formulations.
Martini et. al[42]	Prospective observational	40	Cutaneous hyperchromias	Cosmetic formulation (auto emulsifying bases, polymer, emulsifier, emollients, humectants).	Epidermal pigmentation	Reduction in epidermal pigmentation after 60 days (*p* = 0.0039); hyper-reflective basal cells and increased dermal papillae	RCM can quantity changes in epidermal pigmentation following topical treatments
Piraccini et. al[48]	Prospective observational cohort	52	Brittle nails	​​Solution containing pistacia lentiscus and hyaluronic acid	Total nail thickness, superficial layer of the nail thickness, and the density of the nail.	RCM revealed brighter looking nail plate as well as a clear reduction in canaliform ridges and bright white-pin point structures	Nail strengthener containing Pistacia lentiscus and hyaluronic acid solution improved nail thickness and strength
Berardesca et. al[44]	Prospective observational cohort	40	​​Atopic dermatitis	Shower cream versus shower cream + lotion	SC architecture	Noticeable changes were detected by RCM at 6 weeks, with increased organization and compaction of the SC.	Combination of shower cream and lotion with physiological lipids provided maximum results for restoring skin barrier function and increasing skin hydration.
Rossi et al. [46]	Case series	2	Acne vulgaris	Plasma exeresis	Morphology of pilosebaceous infundibular alterations	At time 0: comedones, papular-pustular lesions, dilated infundibula with thickened bright border At 6 months: disappearance of acne lesions	RCM can be a useful tool in objective evaluation of acne morphology.
Baek et. al[45]	Prospective observational cohort	6	Comedogenicity	Cocoa butter topical cream	Number of microcomedones and follicles	Mean abundance of microcomedones and follicles was higher in the comedogenic group than control at 2 weeks (*p* < 0.0419). Mean value of diameter change was also higher in cocoa butter group at (*p* < 0.0026 at 2 weeks and *p* < 0.0038 at 4 weeks)	RCM can be used to non-invasively determine the comedogenicity of product
Manfredi et. al[47]	Prospective observational study	20	Acne vulgaris	Topical anti-acne product	Morphology of pilosebaceous infundibular alterations	Reductions in infundibular with thickened bright border, dilated infundibula/comedos, inflammatory papules and inflammation (all *p* < 0.05), increase in regular follicles and infundibula (*p* < 0.05) at 6 weeks post treatment	RCM can be utilized to quantitatively evaluate infundibula/comedos

Figure legend: PEG = poly ethylene glycol, RCM = reflectance confocal microscopy, SC = stratum corneum, CALM = café au lait macules, QSAL = Q-switched alexandrite laser, SPF 50 + = sun protection factor 50 +, DEJ = dermal epidermal junction, IPL = intense pulse light, UVB = ultraviolet B, UVA = ultraviolet A, h = hour

**Table 3:** Summary of included studies evaluating RCM for characterization/treatment monitoring in cosmetic indications

Study	Study type	Sample size	Aesthetic condition	Intervention	RCM measure assessed	RCM related results	Key study finding
Richters, et al. [29]	Prospective observational cohort	16 (RCM applied in two additional subjects)	Sensitive skin	Sensitive skin questionnaire including retrospective assessment of four skin sensations and signs vertical single stroke was applied with a commercially available CE marked 1,435 nm non-ablative fractional photo thermolysis home-use laser device Measurements performed in four areas of approximately 1 cm2, at 0.5, 8, 24, and 72 h after stimulus	Peri- and intralesional epidermal proliferation and changes in keratinocyte differentiation	Microthermal zones induced significant changes from the upper (SC) to the deeper dermis, resulting in SC and basement membrane destruction. Damaged capillaries and dermal-epidermal clefting noted after 0.5 h, along with low reflecting area and evident material characteristic changes afterward. Re-epithelialization, keratinocyte migration, and collagen changes in the papillary dermis occurred after 24 h. In interlesional skin, proliferating keratinocytes and viable epidermal thickness increased at 24 and 72 h.	RCM shows re-epithelialization at 24 and 72 h after laser treatment and collagen changes were observed; this characterization helps understanding laser’s effects and applications
Ma, et al. [30]	Cross-sectional observational study	166	Sensitive skin	Sensitive skin questionnaire with 28 factors and RCM with high-resolution horizontal and vertical mapping, followed by lactic acid sting test	Sensitive skin markers: parakeratosis, honeycomb pattern, spongiform edema, and dermal papillae	Sensitive and healthy control groups had disarranged honeycomb pattern and spongiform edema, with no distinct pattern for damaged dermal papilla rings (*p* < 0.05); honeycomb structure depth differed between the sensitive group and healthy control group (*p* < 0.05), while epidermal thickness showed no significant variance (*p* < 0.05)	‘Epidermal honeycomb structure’ and ‘spongiform edema’ may be used as new skin signs of RCM evaluation of sensitive skin effectively
Maier, et al. [34]	Case report	1	Permanent make-up treatment reaction	In vivo imaging followed by punch biopsy	Pigment detection/localization, morphological structure of granulomatous reactions	RCM reveals bright clusters in the superficial dermis, revealing darker centers corresponding to macrophage nuclei, and granuloma formation as clustered bright structures consistent with pigment-loaded macrophages	RCM detects pigment load and structure of granulomatous reactions due to permanent makeup tattooing
Ciardo et al. [25]	Methodological study	50	Skin aging	Evaluation of cheekbone area in two different age groups	Shape and clusters of keratinocytes as well as presence and extent of papillary contours	Irregular honeycomb pattern of keratinocytes and mottled pigmentation were significantly higher in elderly (1.20 v 2.24, *p* < 0.05 and 1.16 v 1.80, *p* < 0.05, respectively).	RCM can be used to detect key signs of aging.
Masuda, et al. [24]	Cross-sectional observational study	103	Skin aging	SSMT of replicas from cheeks of different age groups taken after face washing and resting for 40 min at 23 °C and 45% relative humidity	Skin surface area and roughness; the area of pores; the area, length, depth and width of skin furrows; and the number of skin ridges	Surface roughness, the area of pores and the depth of skin furrows increased with age; area and length of skin furrows and the number of skin ridges decreased with age	Younger cheeks have small pores and fine SSMT evaluated by RCM
Infante, et al. [12]	Cross-sectional observational study	23	Photoaging	Assessment of sun protection habits and their impact on skin visual characteristics, using high-resolution imaging, ultrasound, RCM	Epidermis and papillary dermis morphology	Thinner stratum corneum in Group A (sun protection) vs. Group B (no sunscreen) (*p* < 0.001) Deeper papilla in Group A than Group B, attributed to sun exposure (*p* = 0.034) Coarse fibers: 30.4%, Collagen huddles: 8.7% Irregular honeycomb pattern observed in 7 out of 13 participants without sunscreen	Sun exposure without photoprotection results in changes in the epidermis structure and collagen fibers regardless of chronological age
Infante, et al. [14]	Cross-sectional observational study	40	Photoaging	Evaluation of sun exposure unintentionally and photoprotection habits followed by RCM and scoring based on authors’ methodology	(SC) reflectance and shape; SG honeycomb pattern and interkeratinocyte reflectance; dermis–epidermis junction density and papillary quality; superior dermis quality and collagen density	Sun-protected upper epidermal layers exhibited superior author-designated scores in morphological categories particularly in the DEJ; photoprotection strongly correlated with improved DEJ and collagen network scores	Sun protection habit is more important than age for better morphological and structural features of the skin
Haytoglu, et al. [9]	Cross-sectional observational study	120	Photoaging	Evaluation of sun-exposed malar area, the anterior aspect of ear lobule, and the sun-protected posterior aspect of ear lobule	Suprapapillary epidermal thickness, mottled pigmentation, irregular honeycomb pattern, furrow depth, polycyclic papillary contours, number of papillae, thin reticulated collagen, coarse collagen structures, huddled collagen, curled bright structures	More frequent irregular honeycomb pattern, polycylic papillary contours, coarse collagen, huddled collagen, curled bright structures with higher epidermal thickness, and furrow depth values in sun-exposed areas; statistically higher thin reticulated collagen, mean epidermal thickness and the number of dermal papillae on the sun-protected posterior aspect of ear lobule (*p* < 0.001)	RCM is a reliable diagnostic technique for evaluation of skin photoaging in cosmetically sensitive areas.
Mercurio, et al. [11]	Cross-sectional observational study	82	Photoaging	Influence of solar exposure and protection habits on skin pigmentation using questionnaires, biophysical measurements, and skin imaging	Epidermal layer thickness and epidermal morphological and structural characteristics	Brazilian skin exhibited a greater occurrence of rete ridge effacement and reduced interkeratinocyte reflectance (*p* < 0.01), along with a significantly higher prevalence of huddled collagen (*p* < 0.01)	RCM evaluation of morphological, structural, and biophysical differences observed between French and Brazilian white skin
Fossa Shirata, et al. [10]	Prospective observational study	44	Photoaging	Morphological and structural changes caused by solar radiation in different age groups	Mechanical properties, pigmentation pattern, morphological and structural changes	Group 1 (18–35 year old): Mottled pigmentation (50%), polycyclic papillae (83%), irregular rings (83%), thin reticulated collagen (83%), coarse collagen structures (33%), huddled collagen (17%) Group 2 (40–60 year old): Irregular honeycomb pattern (33%), mottled peigmentation (83%), polycyclic papillae (33%), irregular and absence of rings (50%), coarsed and huddled collagen (100%), curled bright structures (83%)	RCM findings characterize changes in pigmentation pattern, papilla format, and depletion of thin collagen fibers in photoaging.
Razi, et al. [31]	Case study	4	Photoaging	Facial chemical peeling	Changes in inflammation, melanin, and collagen	All four subjects had inflammatory cells at 5 min and resolved 48 h after treatment. 4 h hours post peeling, melanin intensity decreased in basal DEJ and increased in suprabasal layer. Collagen fragmentation is highest at 48 h	RCM can elucidate mechanism of action of cosmetic procedures including chemical peeling.
Campione, et al. [23]	Proof of concept trial	11	Photoaging	Retinoic acid (0.02%) and glycolic acid (4%) gel	Skin cell modification	Reduction of skin aging (Glogau) score at week 4 (*P* = 0.001) (3.4 ± 0.5 to 2.7 ± 0.6). Reduction in dark spots (40%) and severity of wrinkles (12%)	RCM can assess Glogau, dark spot, and wrinkle severity.
Fossa Shirata, et al. [17]	Randomized controlled clinical trial	60	Photoaging	Clinical efficacy of sunscreen and cosmetic formulations containing ascorbyl tetraisopalmitate and rice peptides	Structural and morphically characteristics of the epidermis and dermis papilla depth	Significant increase in granular layer thickness in treatment groups. Increased number of keratinocytes per area, and mottled pigmentation score changed in one group using combination treatment.	RCM can characterize epidermal layer thickness and papilla depth.
Josse, et al. [41]	Randomized controlled trial	17	Solar lentigo	SPF 30 daily skin cream on one hand	Lentigo pigmentation at basal layer, papillae structure	On non-treated lentigines there was a small increase in melanin contrast over time, whereas a decrease in contrast change in treated lentigines (− 3.8 ± 3.1, *p* = 0.075)	Melanin gives high contrast in RCM images and allows for better visualization of skin lentigines.
Martini, et al.[40]	Cross-sectional observational study	40	Skin pigmentation	Skin characterization and questionnaire for assessment of solar exposure and protection habits	Sequences of confocal sections at areas of interest; cell shape and brightness in the basal cell layer of the lesion and in normal perilesional skin	Low sunscreen use/high sun exposure associated with greater accumulation of melanin in the cells of the basal layer and distributed irregularly among different keratinocytes	Melanin appears white in RCM images due to its high refractive index
Altintas, et al.[26]	Cross-sectional observational study	10	Overweight skin’s cutaneous microcirculation	RCM measurements on the volar aspect of a randomly selected forearm in a fasting state, with subjects adhering to a 48-hour avoidance of drugs and cosmetic products	Dynamic processes of microcirculation (ex. dermal blood flow), density of dermal capillaries, epidermal thickness, epidermal cell size	Inverse correlation of overweight and dermal capillary density (*p* < 0.05) while blood cell flow was positively related (*p* < 0.05) to being overweight; increase of both ET (*p* < 0.05) and cell size (*p* < 0.05)	Overweight skin, is characterized by both impaired microvascular function and remodeling of skin histomorphology compared to lean skin
Casari, et al. [43]	Prospective observational study	10	Irritant skin	Irritation was induced by patch test containing sodium lauryl sulfate, and vitamin E application on affected skin was evaluated	Detached corneocytes, targetoid keratinocytes, keratinocyte shape, exocytosis, polygonal structures, papillary shape, inflammatory infiltrate	In treatment group: Less detached corneocytes (*p* < 0.05), faster decrease in targetoid keratinocytes in treated area (*p* < 0.05), less changes in dermal papillae (*p* < 0.05), more regular shaped keratinocytes, less pentagonal architectural disintegration.	RCM can evaluate irritant skin barrier damage and epidermal cell morphology
Asserin, et al. [13]	Randomized, placebo controlled clinical trial	33	Dry skin	Oral collagen peptide supplementation	Degree of collagen fragmentation following	Intake of collagen supplementation reduces fragmentation by 17.8% at 4 weeks and 31.2% at 12 weeks.	RCM can characterize level of collagen fragmentation.
Maia Campos P, et.al[28]	Prospective observational study	19	Oily skin	Skin characterization using RCM of face, chin and nose	RCM visualization of pores, open and closed comedones	Highest production of sebum in nose region, high number of sebaceous glands in the chin, and high number of pores in the chin.	RCM can evaluate comedones and sebaceous glands

Figure legend: SC = stratum corneum; SG = stratum granulosum; DEJ = dermal-epidermal junction; ET = epidermal thickness, SSMT = Skin surface micro-topography, retinol = RET05, RCM = Reflectance Confocal Microscopy, QSAL = Q-switched alexandrite laser, CALM = café au lait macules

## Healthy skin and film-forming effects

RCM has been utilized to measure the efficacy of various treatments and evaluate their film forming effect. In comparisons of two formulations containing starch and PEG-75 lanolin with and without Spirulina maxima dry extract, RCM found improvement of SC morphology, increased structure of epidermis granular layer, and a reduction in furrow distance was observed 30 min after application of formulation (*p* < 0.001), with no difference in the efficacy of both formulations in promoting film formation [6]. 

In evaluation of gel and emulsion formulations with Kappaphycus alvarezii and Caesalpinia spinosa extracts, RCM demonstrated a reduction of furrow width between islands of keratinocytes on the surfaces of the skin, formation of film on the skin surface, and an increase in skin surface brightness through increased skin stratum corneum reflectance before and after, the emulsion formulation compared to the gel formulation after 1 h of application [7]. 

## Photoaging and sunscreen effects

RCM has been utilized to evaluate physio-mechanical and film-forming properties of sunscreen and biopolymer clinical applications. RCM identified increased SC thickness, increased inter-keratinocyte reflectance (both *p* < 0.05) after application, and improvement in furrow morphology and furrow reduction [8]. 

RCM has also been utilized for the evaluation of photoaging. Examination of the sun-exposed malar area, the anterior aspect of the ear lobule, and the sun-protected posterior aspect of the ear lobule indicated a higher frequency of irregular honeycomb patterns, polycyclic papillary contours, coarse collagen, huddled collagen, and curled bright structures in sun-exposed areas, correlating with increased epidermal thickness and furrow depth. Conversely, the sun-protected posterior aspect of the ear lobule exhibited statistically higher values for thin reticulated collagen, mean epidermal thickness, and the number of dermal papillae (*p* < 0.001) [9]. RCM can identify morphological and structural changes caused by solar radiation in different age groups. In one study, patients aged 18–35 had mottled pigmentation (50%), polycyclic papillae (83%) and thin reticulated collagen (83%) whereas in 40–60 year olds it had huddled collagen (100%), curled bright structures (83%) and irregular/absence of rings (50%) [10]. 

RCM has identified differences in skin morphology in areas of sun exposure and sun protection habits, thereby identifying differences in photoaging tendencies. For example, Brazilian skin with increased solar exposure exhibited a greater occurrence of rete ridge effacement (*p* < 0.05) and huddled collagen (*p* < 0.01) and reduced inter-keratinocyte reflectance (*p* < 0.01) under RCM than French skin [11]. Furthermore, RCM has demonstrated that skin with sun protection has a significantly thinner stratum corneum compared to the skin not experiencing sunscreen use (*p* < 0.001), along with a more defined and deeper papilla than without sun protection (*p* = 0.034) and with an irregular honeycomb pattern, indicating morphological damage attributed to sun exposure [12]. Upper epidermal layers with sun protection have exhibited better morphological and structural skin features than deeper layers, particularly for the dermal–epidermal junction, and photoprotection habits have shown strong correlations with improvements for the dermal–epidermal junction and collagen networks [13]. RCM has hence revealed that sun protection may play a more crucial role than age in achieving enhanced morphological and structural skin features [14]. 

RCM can be utilized to assess treatment of photoaged skin. In a study of 40 patients receiving essential oil formulations, RCM displaced increases in epidermal thickness (*p* = 0.004) and papillary depth (*p* = 0.03) [15]. Similarly, irradiation with UVA and UVB in protective cream treated areas compared to unprotected skin with RCM indicated significant changes in bright epidermal cells (*p* = 0.007), parakeratosis (*p* = 0.001), whereas areas not photoprotected had absence of spongiosis, microvesicles, and blood vessel dilation (*p* < 0.05) [16]. RCM can be used to evaluate efficacy of sunscreen and cosmetic formulations, for example one study found a formulation containing ascorbyl tetraisopalmitate and rice peptides led to increased granular layer thickness and increased keratinocytes per area [17]. 

## Aging skin

RCM has been utilized to evaluate structural changes of aged neck skin treated with laser skin resurfacing treatments. Findings include improvement in dyschromia (*p* = 0.0002), and wrinkles (*p* = 0.0004) with no changes in laxity [18]. Nasolabial folds treated with hyaluronic acid dermal fillers show a 32% increase in height of the JED at 12 months (*p* < 0.001) [19]. 

The appearance of the epidermis appearance following 0.5% cosmetic retinol application on alternate days and indicated a decrease in the SC thickness (*p* < 0.02), with a 5.3% overall decrease in epidermal thickness, along with an increase in the presence of fibrillar collagen at weeks 8 and 12 (all *p* ≤ 0.0469) and a decrease in the presence of huddled collagen at week 12 (*p* < 0.0313) [20]. 

In evaluation of the efficacy of daily moisturizer with high UVB and UVA photoprotection, RCM has demonstrated reduced appearance of UV damage relating to spongiosis, microvesicles, sunburn cells and blood vessel dilation [16]. RCM evaluation of a topical antioxidant serum containing 15% L-ascorbic acid, neohesperidin, Pinus pinaster bark, tocopherol, and hyaluronic acid revealed a significant increase in SC thickness (*p <* 0.047*)* and a reduction of basal membrane pigmentation (*p <* 0.005) [21]. 

RCM can detect changes in papillary dermis following anti-aging peptide topical applications. The depth of papillary dermis was reduced from 108 to 63 μm (R²=0.517) with increases of + 13.2% in dermal fiber circumference (*p* < 0.01). Additionally, reductions in subepidermal low-echogenic band by 11% in the inner face (*p* < 0.01) and 14.4% in the external surface of the forearm (*p* < 0.01) were noted following anti-aging cream application [22]. In another study evaluating retinoic acid and glycolic acid gels, RCM identified changes in Glogau scoring, reduction in dark spots and severity of wrinkles [23]. 

RCM has also been used to evaluate morphological and structural characteristics of the skin in aging. With increasing age, RCM found a noticeable rise in surface roughness, the area of pores and the depth of skin furrows. Conversely, the area, length of skin furrows, and number of skin ridges, exhibited a decrease with age. RCM also revealed smaller pores and finer skin surface micro-topography (SSMT) in younger individuals [24]. In another study RCM has also found a significantly more irregular honeycomb pattern of keratinocytes and mottled pigmentation in the elderly (1.20 v 2.24, *p* < 0.05 and 1.16 v 1.80, *p* < 0.05, respectively) when used to evaluate keratinocyte and papillary contours in order to determine key signs of aging [25]. 

## Cutaneous microcirculation

RCM measurements conducted on the volar aspect of a randomly selected forearm in a fasting state in overweight skin also have revealed an inverse correlation between the condition of being overweight and dermal capillary density (*p* < 0.05), while blood cell flow have showed a positive relationship (*p* < 0.05) to being overweight. The increase in both epidermal thickness (*p* < 0.05) and cell size (*p* < 0.05) have indicated significant changes in human skin histomorphology in overweight individuals [26]. 

### Skin dryness

RCM showed presence of micro-scales and surface irregularity in dry skin, with decreases in scale score, furrow morphology, and epidermal irregularity scores in moisturizer applied areas (*p* < 0.001). However, there were increases in inter-keratinocyte reflectance in applied areas [27]. On the other hand, Skin characterization of oily skin of the face, chin, and nose reveal higher sebum production in the nose, high sebaceous glands in the chin and high pores in the chin, highlighting the usefulness of RCM in evaluating comedones and sebaceous glands in skin [28]. 

## Sensitive skin

In sensitive skin, following treatment with a 1,435 nm non-ablative fractional photothermolysis home-use laser device, RCM played a crucial role in visualizing re-epithelialization at 24 and 72 h after treatment, along with observing collagen changes; in intralesional skin, the number of proliferating keratinocytes and viable epidermal thickness increased at 24 and 72 h [29]. Disarranged epidermal honeycomb structure and spongiform edema (*p* < 0.05) have also proved to serve as new skin signs for RCM evaluation of sensitive skin [30]. RCM evaluation of facial chemical peeling reveals changes in inflammatory cells after treatment with decreased melanin intensity in basal dermal-epidermal junction with increased collagen fragmentation at 48 h, revealing the utility in RCM for highlighting the mechanism of action of certain cosmetic procedures [31]. 

## Abdominal striae

In stretch marks, RCM demonstrates an abnormal elongation in the shape of dermal papillae at the dermal epidermal junction in stretch compared to normal skin. At the dermal level, RCM indicates a parallel alignment of collagen bundles, in contrast to the cross-linked appearance observed in normal collagen. Following bipolar radiofrequency treatment, RCM indicates dermal papilla becoming rounder and collagen fibers exhibiting a shift from a parallel disposition to a more reticulated pattern. Significantly, these RCM features are found differed in the combination-treated and fractional bipolar RF-only treated quadrants compared to the control, (*p-value* = 0.02) [32].

## Pigmentation disorders

### Pigmented lesions

After three sessions of Q-switched alexandrite laser (QSAL) for the reduction of café au lait macules (CALMs), RCM revealed that CALMs with irregular border presented with shorter ret pegs and less papillae (*p* < 0.05) following laser treatment, in comparison to the smooth bordered lesions [33]. Results also indicated an inverse relationship between response treatment and length/abundance of ret pegs along papillae. RCM can detect pigment load and structure of granulomatous reactions secondary to cosmetic procedures, such as makeup tattooing, revealing bright clusters in superficial dermis and dark centers for macrophage nuclei [34]. 

### Hyperpigmentation

In evaluation of the efficacy of skin whitening serum in combination with spot-preventing SPF50 + sunscreen in reducing the appearance of melasma hyperpigmentation, RCM detected improvement in melasma through detection of reduced inflammatory infiltrate from baseline to the end of treatment, as well as a reduction in melanophage and dendritic cell abundance [35]. Similarly, reduction in the cobblestone pattern and dendritic cell presence was associated with treatment success post treatment (*p* < 0.001) [36]. 

In treatment of solar lentigo hyperpigmentation with a dermocosmetic lightening product, RCM showed a statistically significant difference in papillary brightness (*p* < 0.03), as well as papillary contrast (*p* < 0.03), ultimately suggesting the efficacy of dermocosmetic lightening products in reducing solar lentigo hyperpigmentation [37]. By detecting focal peri adnexal brightness, RCM has also performed treatment monitoring of solar lentigo by evaluating changes in pigmentation in keratinocytes and DEJ following application of a depigmenting agent [38]. SPF containing daily creams have shown to decrease melanin contrast in treated areas (*p* = 0.075), and RCM has been identified as a tool to allow better visualization of skin lentigines [39]. 

Additionally, RCM analysis of confocal section sequences at areas of interest, focusing on cell shape and brightness in the basal cell layer of cutaneous hyperchromic lesions and in normal perilesional skin, has revealed that low sunscreen use, and high sun exposure are associated with a greater accumulation of melanin in the cells of the basal layer, distributed irregularly amongst different keratinocytes [40]. RCM provided a high contrast of melanin and morphology of skin lentigines, allowing for a high-resolution visualization of pigmentary changes in sunscreen and non-sunscreen applied surfaces [41]. It was also employed to quantify changes in epidermal pigmentation (*p* = 0.0039), following cosmetic applications, and show hyper-reflective basal cells around non reflective papillary dermis with increases in dermal papillae [42]. 

## Other skin conditions

### Irritant contact dermatitis

RCM can identify skins of skin irritation including corneocyte, keratinocyte and papillary shape changes. It has also been used to evaluate the preventative effect of topical vitamin E application in decreasing detached coenocytes, reducing damaged keratinocytes, and maintaining regular dermal papillae (all *p* < 0.05) [43]. In atopic dry skin, RCM revealed increased organization and compaction of stratum corneum (SC) with use of both shower cream and lotion at six weeks [44]. 

### Comedogenicity detection

Following the utilization of cocoa butter to induce microcomedones, has shown an increase in the mean abundance of microcomedones and follicles (*p* < 0.0419), the mean value of diameter change at two weeks (*p* < 0.0026) and at four weeks (*p* < 0.0038) [45]. 

### Acne Vulgaris

RCM imaging allows for visualization of the morphology of the pilosebaceous unit, and demonstrates reductions in comedones, papulopustular, and dilated infundibula with bright borders following plasma exeresis treatment in active acne [46]. These changes can also be described quantitatively, and RCM evaluating acne post topical treatment showed reductions in inflammatory papules and inflammation (*p* < 0.05), as well as increase in regular follicles and infundibula (*p* < 0.05) at 6 weeks post treatment [47]. 

### Nails

RCM has also been used to characterize in evaluated treatments for improving nail strength. Following application of water-based nail-strengthening solution containing pistacia lentiscus and hyaluronic acid for nail brittleness, RCM results signified a brighter more regular looking nail plate as a well as a clear reduction in canaliform ridges and bright white-pin point structures [48]. 

## Discussion

The prevalence of cosmetic dermatology consultations and procedures has been increasing, with over 11 million surgical/nonsurgical procedures conducted in 2013 [49]. In cosmetic contexts, RCM has primarily been investigated for characterization and treatment monitoring purposes. RCM allows for visualization of furrow and stratum corneum morphology, epidermal changes, dermal papillae and collagen, and pigmentation and melanin distribution. It has clinical utility in evaluation of skin lesions and treatment efficacy, along with characterization of photodamage, aging, melasma, comedones, hyperpigmentation, and striae distensae, before and after treatments.

**Fig. 1:**
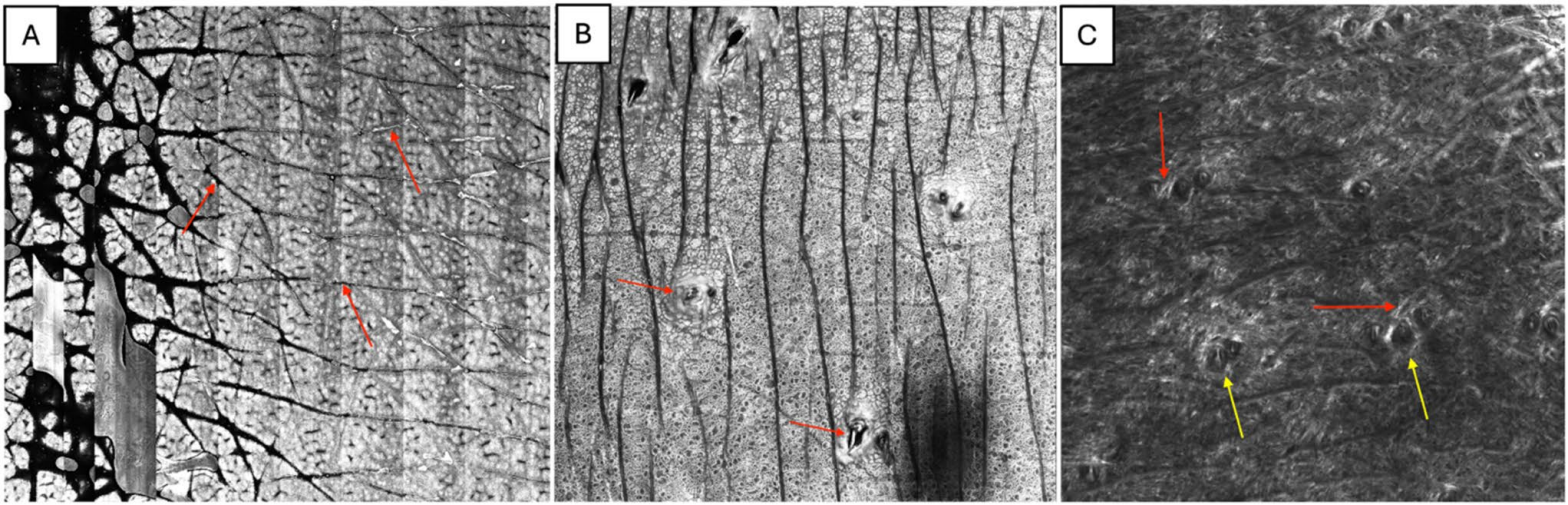
Reflectance confocal microscopy imaging of normal skin. RCM of normal skin at multiple levels. (**A**) RCM image at the level of the epidermis. The hallmark feature, regular honeycombing, representing epidermal keratinocytes, can be seen along with skin furrows (red arrows). (**B**) RCM image at the level of the dermal-epithelial junction. Hyperreflective rings representing dermal papillae can be appreciated. Due to the skin's innate undulations, a part of the overlying epidermis with a honeycomb pattern can be seen in part of the skin (red arrows), with a hair follicle at the center. (**C**) RCM image at the level of the superficial dermis. Hyperreflective collagen bundles are visible (red arrows), along with a few scattered hair follicles (yellow arrows)

RCM is a non-invasive imaging tool that may improve the diagnostic accuracy and track treatment progress. RCM imaging of the epidermis provides real time visualization of epidermal keratinocytes, the dermal-epidermal junction, superficial dermis and collagen bundles with hair follicles (Fig. 1).

**Fig. 2:**
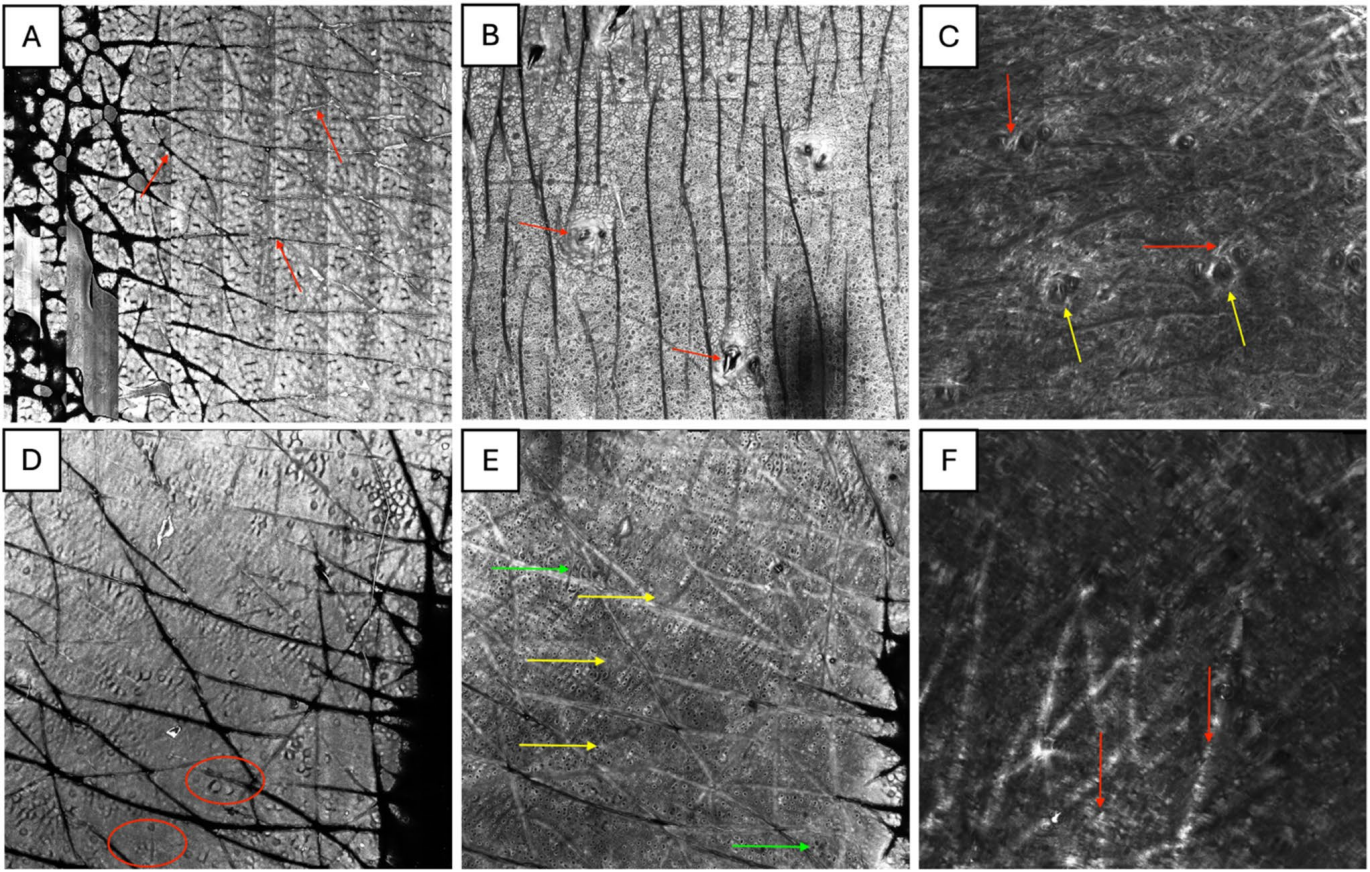
Reflectance confocal microscopy imaging findings of normal skin compared to photoaged skin. RCM of normal and aging skin at multiple levels. (**A**) RCM image at the level of the superficial papillary dermis in a 23-year-old. Hyperreflective collagen bundles are visible (red arrows), along with a few scattered hair follicles (yellow arrows). (**B**) RCM image at the level of the dermal-epithelial junction in a 23-year-old. Hyperreflective rings representing dermal papillae can be appreciated. Due to the skin’s innate undulations, a part of the overlying epidermis with a honeycomb pattern can be seen in part of the skin (red arrows), with a hair follicle at the center. (**C**) RCM image at the level of the epidermis in a 23-year-old. The hallmark feature, typical honeycombing, representing epidermal keratinocytes, can be seen along with skin furrows (red arrows). (**D**) RCM image at the level of the superficial papillary dermis in a 67-year-old. Hyperreflective coarse collagen bundles are visible (red arrows). The overall density of collagen bundles is decreased. (**E**) RCM image at the level of the epidermis in a 67-year-old. Several patches of atypical honeycombing can be seen, consisting of polygonal keratinocytes with ill-defined cell borders (red circles). (**F**) RCM image at the level of the dermal-epithelial junction in a 67-year-old. Mottled pigmentation (yellow arrows) and polycyclic papillary contours rings (green arrows) can be seen

In contrast to normal skin, RCM can visualize changes in photoaged skin including hyperreflective collagen bundles, atypical honeycombing, mottled pigmentation and ill-defined dermal-epidermal cell borders **(**Fig. 2**)**.

Reflectance confocal microscopy has become an increasingly helpful adjunctive tool to the clinical exam; through creating horizontal images of the skin at the cellular and subcellular level, a near histological resolution of the skin can be evaluated at bedside. RCM has been utilized in various diagnostic algorithms, including in assessment of melanocytic and non-melanocytic lesions with high pooled sensitivities of up to 92–95%. Additionally, RCM criteria have been developed to evaluate features of basal cell carcinoma and melanoma based off of dermoscopy and confocal features alone [50]. This poses a significant benefit in improving diagnostic accuracy, limiting delays in treatment initiation, and potentially limiting the need for biopsy in cosmetically sensitive areas [50]. RCM can similarly guide treatment clinically: mapping of large tumors may help identify defining margins for correct surgical excision, and treatment monitoring may assess treatment response and/or recurrent disease [51]. Large scale studies evaluating its use in cosmetic use has not been as extensively studied, but we find its clinical utility in treatment monitoring and skin characterization to be useful in assessing treatment response in a variety of aesthetic skin applications.

Evaluations of cosmetic improvements in skin has primarily been limited to clinical evaluations with/without dermoscopy. The use of biopsy, which can illustrate histo-structural changes at the cellular level, is limited in cosmetic indications due to risk of scarring, pain, bleeding, and infection [52]. Results from our study suggest that RCM is effective in illustrating changes in cosmetic use at the level of the epidermis and dermis; an important finding that provides another layer of objective evaluation of the skin when biopsy is not indicated or practical (in aesthetic skin application). Future large-scale studies are needed to prospectively analyze the efficacy of RCM treatment monitoring in cosmetic applications including assessing treatment duration, product selection, and condition of use.

The rising popularity of treatment for aesthetic conditions has notably increased as individuals seek personalized solutions to address cosmetic and aesthetic concerns.

One notable issue is the limited availability of objective comparator studies, making it difficult for both patients and practitioners to assess the efficacy of various cosmetic procedures comprehensively. Furthermore, the field is confronted with the task of addressing elevated patient standards and expectations, which encompass a range of preferences regarding physical appearance influenced by cultural, historical, and media factors, and evolving over time [53, 54]. RCM seeks to address such obstacles by offering a non-invasive means to comprehensively assess treatment efficacy, providing detailed insights that enable tailored interventions for diverse patient experiences.

There are some limitations to RCM for cosmetic purposes. RCM has limited depth penetration of 350–400 μm with decreasing resolution below 100–150 μm, affecting visualization of the skin deeper than the papillary dermis. Utilization of RCM on non-flat surfaces poses an application challenge, and imaging of conditions that typically obscure light as ulceration, bleeding, and crusting, have limited visualization. The high cost of RCM machines and limited practitioners trained in RCM utilization (approximately 4–6 months of training) and image interpretation also limit its broad clinical use [55]. RCM takes significantly longer than dermoscopy and is not a replacement but rather serves as an adjunct for screening and treatment monitoring. Future developments in RCM technology may include development of greater depth of imaging, utilization of color contrast technology, and integration with artificial intelligence [55–58]. 

## Conclusion

RCM is a non-invasive high resolution imaging tool that provides real time, visualization of histologic changes in the skin. RCM has been utilized for cosmetic purposes, primarily to characterize epidermal and dermal structural changes in furrow morphology, epidermal thickness, melanin, dermal-epidermal junction changes, keratinocyte structure, dermal papillae, collagen changes, ridge effacement, and inter-keratinocyte reflectance. Further histologic evaluation may aid in clinical diagnoses and identification of morphologic changes to identify new treatment developments. Treatment monitoring has been successfully conducted using RCM for various skin conditions, such as facial photo damage, comedogenicity, pigmented lesions, melasma, brittle nails, solar lentigo, acne, and abdominal striae following topical treatments. RCM may provide a non-invasive real time visualization of changes which guide treatment selection and treatment duration depending on changes at the cellular level. High cost and education regarding RCM utilization and imaging interpretation are important considerations. Future studies should evaluate larger patient cohorts with a variety of skin conditions and comment on diagnostic efficacy of RCM in cosmetic conditions.

## Data Availability

No datasets were generated or analysed during the current study.
